# Huge intrathoracic desmoid tumor

**DOI:** 10.4103/1817-1737.53350

**Published:** 2009

**Authors:** Majdi Ibrahim, Hasan Sandogji, Abdullah Allam

**Affiliations:** *Department of Thoracic Surgery, King Fahad Hospital, Almadinah Almunawarah, Saudi Arabia*

**Keywords:** Chest wall, desmoid, fibromatosis, intrathoracic, tumor

## Abstract

Desmoid tumors are soft-tissue neoplasms arising from fascial or musculo-aponeurotic structures. Most reported thoracic desmoid tumors originate from the chest wall. However, intrathoracic desmoid tumors are rare. We present a case of a 35-year-old male patient complaining of mild shortness of breath. The patient was diagnosed to have a huge intrathoracic desmoid tumor, which was successfully resected.

Desmoid tumors are soft-tissue neoplasms arising from fascial or musculo-aponeurotic structures.[1,2] Most reported thoracic desmoid tumors originate from the chest wall. However, intrathoracic desmoid tumors are rare.[3] They are classified as benign as they do not metastasize.[4] Desmoid tumors can, however, exhibit rapid local growth, and clinically they can mimic sarcomas. Their histological appearance can also resemble some malignant neoplasm such as low-grade sarcomas, rendering the differential diagnosis difficult.[5] Desmoid tumors account for approximately 3.5% of fibrous tumors, 0.3% of all solid tumors[6] and only 0.03% of all the neoplasms.[7] Chest wall desmoids account for approximately 20% of all desmoid tumors. Patients with these lesions are often asymptomatic and thus commonly present with lesions greater than 10 cm in size.[8]

## Case Report

A 35-year-old male patient was admitted to the emergency room complaining of mild shortness of breath of 12 weeks’ duration. He had history of road traffic accident about 1 year back, with unilateral lower limb fracture. Otherwise, the history was unremarkable.

**Figure 1:**
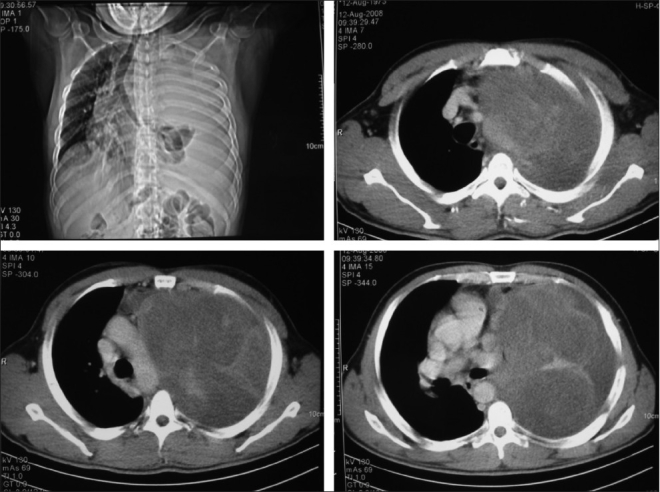
CT scan of the chest — Huge left intrathoracic mass

Clinical examination of the patient showed stable vital signs, one small left supraclavicular lymph node, trachea shifted to the right and decreased air entry on the left side with dull percussion note; otherwise, no abnormality was detected. Blood work-up did not show significant changes. Chest x-ray was done, and it showed homogenous opacity on left hemi-thorax and shifting of the mediastinum to the right. So, CT scan of the chest was done, and it revealed huge left-side intrathoracic mass pushing the mediastinum to the right side, with complete collapse of the left lung [Figure 1]. Fine-needle aspiration of the lymph node was inconclusive. So, incisional biopsy of the intrathoracic mass was done, which showed desmoid tumor.

**Figure 2:**
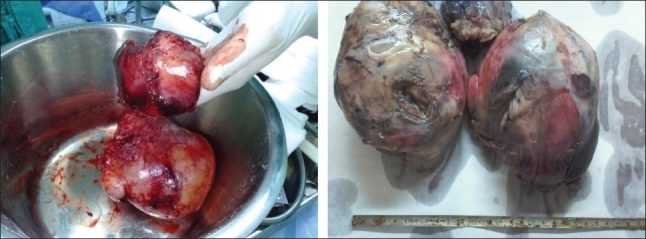
Huge intrathoracic desmoid tumor

**Figure 3:**
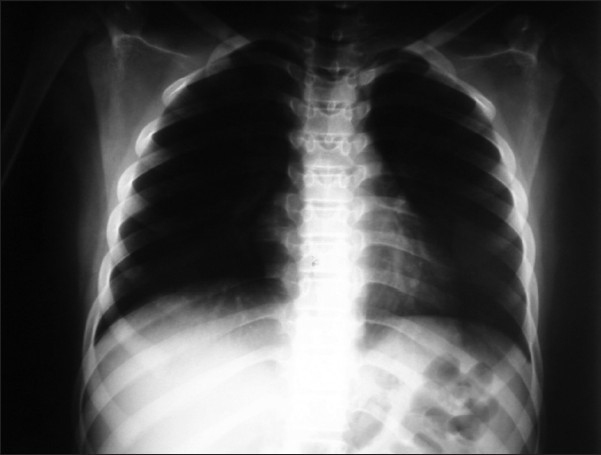
Complete inflation of the left lung

The patient was prepared for surgery, posterolateral thoracotomy was done and about 20 × 15 × 20 cm lobulated intrathoracic tumor was found. The tumor was attached to the apicolateral portion of the chest wall near the brachial plexus; so we excised the tumor completely except a very small part which was adherent to the area of brachial plexus, and decortication of the left lung was done [Figure 2]. The histopathological study of the mass confirmed the diagnosis. The postoperative recovery period went smoothly and the left lung completely expanded [Figure 3]. The patient was referred to our oncologist, who referred the patient to the oncology center in Jeddah for completion of treatment. The patient has not come for follow-up in our hospital till now.

## Discussion

John Macfarlane first described desmoid tumors in 1832.[9] The overall incidence of desmoid tumors is approximately 2–4 cases per million of the population per year.[10] They are rare tumors, which were given different names: Desmoid fibroma, aggressive fibromatosis, desmomas, and desmoplastic fibroma. Currently the name sarcoma of low-grade malignancy[12] or aggressive fibromatosis[6,11] is preferred due to its vulnerability to local invasion and frequent recurrences even after complete surgical resection.[9]

They most often arise from the musculo-aponeurosis of the abdominal wall.[13] Common sites of extra-abdominal desmoid tumors include the extremities, head and neck, and chest wall. The incidence of chest wall desmoid tumor has been reported to be 10% to 28%.[1] Although desmoid tumors of the chest wall account for approximately 20% of all desmoid tumors, only 26 case reports (including our case) of intrathoracic desmoid tumors have appeared in the English literature.[10,14,15]

Several pathophysiological hypotheses are suggested.[16] Abnormal scarring secondary to previous surgery[6,17–21] or chest trauma[22,23]; hormonal factor, particularly estrogen; genetic (familial) predisposition, in relation to clonal abnormalities carried on Y chromosome, or the long arm of fifth, which is related to chromosome playing Gardner's syndrome. As many as 33% to 38% of patients with Gardner's syndrome develop desmoid tumors, but only 2% of patients with desmoid tumors have Gardner's, syndrome or other pathology (familial adenomatous polyposis, osteomas and other soft-tissue neoplasms)[16,24–28] or abnormalities in connective tissue synthesis. Symptoms are rare and result mainly from the local mass effect of tumor encroachment on vital structures or erosion of adjacent bone or joint tissue.[13]

Complete resection of the tumor with a clear surgical margin is currently the mainstay of curative treatment for desmoid tumors. The recurrence rate is high and varies from 29% to 54% in some reported series.[29] Regular follow-up imaging is mandatory even when surgical margins are free of tumors. Re-excision is recommended for local recurrent disease. Other treatment methods, including radiation, chemotherapy, c-AMP modulation, estrogen, and prostaglandin inhibition, have been tried with varying success.[30]
